# Prognostic impact of lymphovascular and perineural invasion in squamous cell carcinoma of the tongue

**DOI:** 10.1038/s41598-023-30939-8

**Published:** 2023-03-07

**Authors:** Qiongling Huang, Yanjun Huang, Chunhui Chen, Yizheng Zhang, Jiao Zhou, Chengke Xie, Ming Lu, Yu Xiong, Dage Fang, Yubin Yang, Weipeng Hu, Feng Zheng, Chaohui Zheng

**Affiliations:** 1 Department of Otolaryngology, the Second Affiliated Hospital, Fujian Medical University, Quanzhou, 362000 Fujian China; 2 Department of Neurosurgery, the Second Affiliated Hospital, Fujian Medical University, Quanzhou, 362000 Fujian China; 3 Department of Gastroenterology, the Second Affiliated Hospital, Fujian Medical University, Quanzhou, 362000 Fujian China; 4 Department of Pathology, the Second Affiliated Hospital, Fujian Medical University, Quanzhou, 362000 Fujian China

**Keywords:** Cancer, Neuroscience, Biomarkers, Diseases, Medical research, Oncology, Risk factors

## Abstract

This study aimed to investigate the prognostic impact of lymphovascular and perineural invasions in patients with squamous cell carcinoma of the tongue who received surgery-based treatment at our institution between January 2013 and December 2020. Patients were divided into four groups based on the presence of perineural (P−/P +) and lymphovascular invasions (V−/V +): P–V−, P–V + , P + V−, and P + V + . Log-rank and Cox proportional hazard models were used to evaluate the association between perineural /lymphovascular invasion and overall survival (OS). Altogether, 127 patients were included, and 95 (74.8%), 8 (6.3%), 18 (14.2%), and 6 (4.7%) cases were classified as P–V−, P–V + , P + V−, and P + V + , respectively. Pathologic N stage (pN stage), tumor stage, histological grade, lymphovascular invasion, perineural invasion, and postoperative radiotherapy were significantly associated with OS (p < 0.05). OS was significantly different among the four groups (p < 0.05). Significant between-group differences in OS were detected for node-positive (p < 0.05) and stage III–IV (p < 0.05) cases. OS was the worst in the P + V + group. Lymphovascular and perineural invasions are independent negative prognostic factors for squamous cell carcinoma of the tongue. Patients with lymphovascular and/or perineural invasion may have significantly poorer overall survival than those without neurovascular involvement.

## Introduction

**Figure 1:**
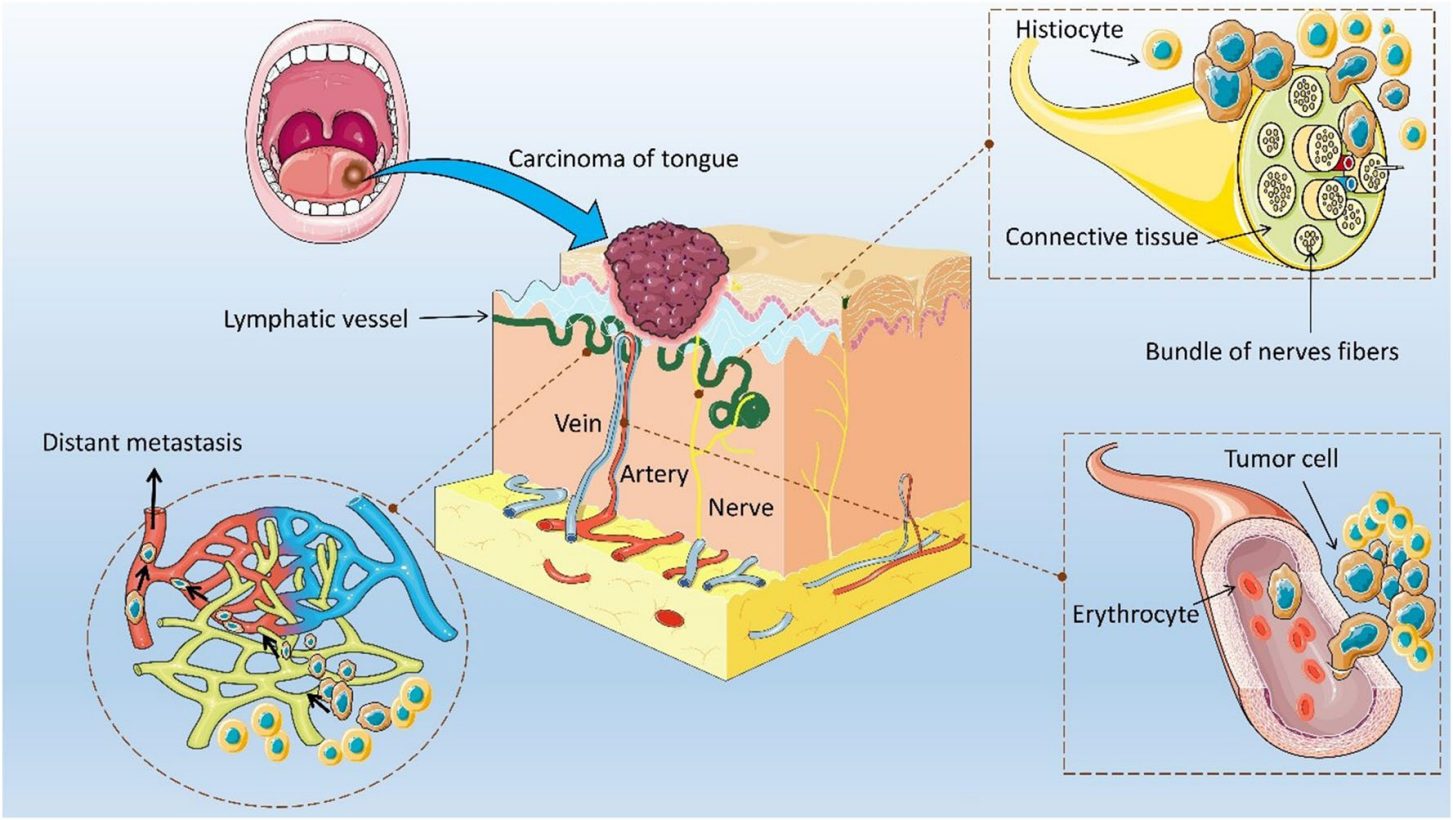
Pathways of tumor cells invading the nerves and vessels. Tumor cells are transmitted indirectly via lymphatic or lymphatic vein exchange channels rather than directly via blood vessels. LVI refers to the localization of tumor cells in the vascular lumen or local adhesion to the vascular wall. PNI refers to cells being close to the nerves and involves 33% of tumor cells or the invasion of three layers of the nerve sheath. A black and thickened arrow in the figure indicates the direction of tumor cell invasion. *PNI* perineural invasion, *LVI* lymphovascular invasion.

Oral cavity cancer is the 16th most common malignant neoplasm worldwide, with approximately 355,000 new cases and approximately 177,000 cancer-related deaths annually^1–3^. Cancer of the anterior two-thirds of the tongue accounts for approximately 37% of newly diagnosed cases of malignancy involving the oral cavity^4^, and approximately 84–97% of tongue cancers are squamous cell carcinomas^5,6^. Over the past decade, the mortality rate of squamous cell carcinoma has been high^7^. Approximately 60% of the five-year survival rate of patients with oral squamous cell carcinoma (OSCC) is unoptimistic despite advances in diagnosing and treating oral cavity cancer^8,9^. Oral cavity cancer is typically diagnosed when it is relatively advanced with a poor prognosis owing to the lack of public awareness; however, this disease has gradually attracted the public’s attention in recent years^10^. The concept of perineural invasion (PNI) and lymphovascular invasion (LVI) has often been discussed in research on OSCC^11–14^. The pathways of tumor cells invading the nerves and vessels are shown in Fig. 1.

Cruveiheir first described PNI in head and neck cancer in 1835^6^. OSCC is a neurotropic malignancy in which neurotropism is a significant feature representing tumor cells and stroma affinity to the surrounding nerve tissue^15^. The incidence rates of PNI in tongue cancer and buccal mucosa carcinoma were approximately 28.3% and 16%, respectively, and this may be attributed to the rich nerve distribution in the tongue^16^. LVI is defined as tumor embolism within the space in which tumor cells invade the vascular wall and/or that in which endothelial cells are arranged^17^. Tumor cells are transmitted indirectly via lymphatic or lymphatic vein exchange channels rather than directly via blood vessels^18^. Although LVI and PNI are widely used as indicators of aggressive tumor cells, there is no definite report of LVI and PNI in squamous cell carcinoma of the tongue (SCCT)^19,20^. The staging of SCCT affects risk stratification, which is the first step toward personalized treatment^21^. The American Joint Committee on Cancer (AJCC) tumor node metastasis is widely available, but whether LVI and PNI can be used as additional staging criteria remains uncertain^2,22,23^. Therefore, this study aimed to investigate the prognostic impact of LVI and PNI in patients with SCCT to establish a complete risk classification.

## Methods

### Patient characteristics

This retrospective study included patients with SCCT who underwent surgery-based treatment at our institution between January 2013 and December 2020. Patients without clinical evidence of lymph node metastasis (N−) usually underwent selective neck dissection, whereas those with lymph node metastasis (N +) underwent modified radical neck dissection^24,25^. Clinicopathological data, including histopathology and surgical records, were retrieved from the patient’s medical records, and the follow-up time was set from the date of surgery to death, loss of visit, or May 2022. Given that the etiology and prognosis of squamous cell carcinoma in the posterior part of the tongue are different from those of the anterior part, only the anterior two-thirds of the tongue was studied in the present research. The inclusion criteria were (1) diagnosis of SCCT based on preoperative imaging and postoperative pathology; (2) no preoperative chemotherapy, radiotherapy, immunotherapy, or endocrine therapy; (3) first onset and treatment occurring during the study period; (4) no distant metastasis detected before surgery; and (5) complete clinical and pathological data. Patients were excluded from the study if they had incomplete records, were lost to follow-up, or had concomitant malignant tumors or a malignant tumor history.

This study was carried out following the Code of Ethics of the World Medical Association (Declaration of Helsinki) for experiments involving humans that were further updated, and the protocol was reviewed and approved by the Medical ethics committee of the Second Affiliated Hospital of Fujian Medical University(no. 274, 2022). All procedures conducted in studies involving human participants met the ethical standards of the Institutional Research Committee. Informed consent was obtained from all patients. All methods were performed in accordance with the relevant guidelines and regulations.

### Clinicopathological features

The eighth edition of the American Joint Committee on Cancer (AJCC) tumor node metastasis (AJCC-TNM 8th) was used to stage all the patients with SCCT. The pathologists at our institution examined the surgical specimens by using the AJCC criteria in a blinded manner. The pathological sections were stained with hematoxylin and eosin to analyze histopathological parameters, including histological grade, staging, pathologic T stage (pT stage), pathologic N stage (pN stage), LVI, and PNI. Patients with LVI, PNI, or lymph node metastasis should undergo postoperative radiotherapy (PORT); hence, PORT was also analyzed as a prognostic factor in our study. The patients were divided into four groups based on the presence of LVI (V−/V +) and PNI (P−/P +): P–V−, P–V + , P + V− and P + V + .

### Statistical methods

Fisher’s exact and the chi-square tests were used to determine differences in clinical features among the four groups. Pearson’s chi-square test was used to identify the correlation between clinicopathological features and overall survival (OS). OS was defined as the time from surgery to death, loss of visit, or May 2022. The Kaplan–Meier method was used to plot univariate survival curves, and a log-rank test was used to determine the statistical differences. Cox proportional hazard modeling was used to estimate the independent prognostic impact of patient- and tumor-related factors on survival, and hazard ratios (HRs) and 95% confidence intervals (CIs) were used to report the magnitude of the differences and the strength of association^26^. All statistical analyses were performed using SPSS software version 26 (SPSS Inc., Chicago, Illinois, USA). Statistical significance was set at p < 0.05.

## Results

### Clinical parameters

**Figure 2:**
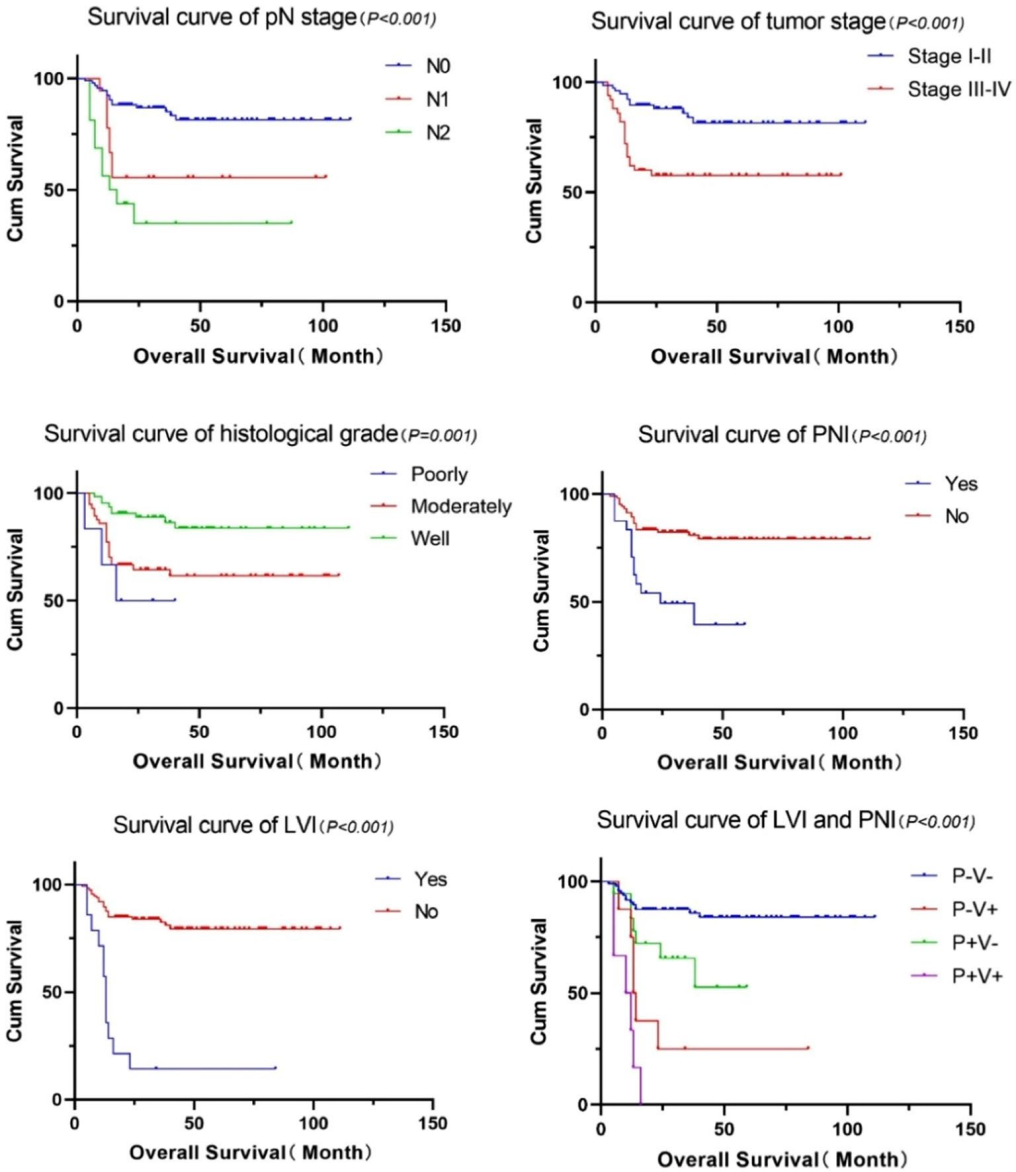
Survival curve of clinical factors and OS. *OS* overall survival.

The study had 219 eligible patients with SCCT who received treatment at our institution from January 2013 to December 2020. However, those who did not have medical records (n = 4), had other malignant tumors (n = 13), were receiving preoperative radiotherapy and chemotherapy (n = 13), refused surgical treatment (n = 46), had postoperative pathology suggestive of other pathological types (n = 4), and were lost to follow-up (n = 12) were excluded. A total of 127 eligible patients were included in the analysis (Fig. S1). Among the 127 patients evaluated in this study, the median age was 57 years (range, 28–87 years), and 68 (53.5%) were male. Table 1 shows the patient demographic information and histological evaluation of the lesional tissue. Significant differences were observed in pT stage, pN stage, and tumor stage. No significant differences among the four groups were detected in sex, age, histological grade, or postoperative radiotherapy. The relationship between these factors and OS is summarized in Fig. 2.

**Table 1:** Patient demographic information and histological evaluation of the lesional tissue.

	P–V−	P–V +	P + V−	P + V +	Total	χ^2^	p-value
Gender							
Female	45 (35.4%)	4 (3.1%)	8 (6.3%)	2 (1.6%)	59 (46.5%)	0.517	0.915
Male	50 (39.4%)	4 (3.1%)	10 (7.9%)	4 (3.1%)	68 (53.5%)		
Age (years)							
< 60	52 (40.9%)	6 (4.7%)	10 (7.9%)	4 (3.1%)	72 (56.7%)	1.493	0.684
≥ 60	43 (33.9%)	2 (1.6%)	8 (6.3%)	2 (1.6%)	55 (43.3%)		
Smoking							
No	73 (57.5%)	5 (3.9%)	13 (10.2%)	4 (3.1%)	95 (74.8%)	1.126	0.771
Yes	22 (17.3%)	3 (2.4%)	5 (3.9%)	2 (1.6%)	32 (25.2%)		
Alcohol							
No	74 (58.3%)	4 (3.1%)	15 (11.8%)	4 (3.1%)	97 (76.4%)	4.003	0.261
Yes	21 (16.5%)	4 (3.1%)	3 (2.4%)	2 (1.6%)	30 (23.6%)		
Pathologic T stage							
T1-2	81 (63.8%)	6 (4.7%)	10 (7.9%)	2 (1.6%)	99 (78%)	15.199	0.002*
T3-4	14 (11%)	2 (1.6%)	8 (6.3%)	4 (3.1%)	28 (22%)		
Pathologic N stage							
N0	78 (61.4%)	3 (2.4%)	12 (9.4%)	0 (0%)	93 (73.2%)	25.835	< 0.001*
N +	17 (13.4%)	5 (3.9%)	6 (4.7%)	6 (4.7%)	34 (26.8%)		
Tumor stage							
I-II	67 (52.8%)	3 (2.4%)	7 (5.5%)	0 (0%)	77 (60.6%)	18.495	< 0.001*
III-IV	28 (22%)	5 (3.9%)	11 (8.7%)	6 (4.7%)	50 (39.4%)		
Histological grade							
Poorly-moderately	44 (34.6%)	7 (5.5%)	8 (6.3%)	4 (3.1%)	63 (49.6%)	5.897	0.117
Well	51 (40.2%)	1 (0.8%)	10 (7.9%)	2 (1.6%)	64 (50.4%)		
Postoperative radiotherapy							
No	83 (65.4%)	6 (4.7%)	12 (9.4%)	6 (4.7%)	107 (84.3%)	6.528	0.089
Yes	12 (9.4%)	2 (1.6%)	6 (4.7%)	0 (0%)	20 (15.7%)		

*P−V−* PNI-negative with LVI-negative, *P–V* + PNI-negative with LVI-positive, *P* + *V−* PNI-positive with LVI-negative, *P* + *V* + PNI-positive with LVI-positive.

*Statistically significant difference.

### One-, three-, and five-year survival rates 

**Figure 3:**
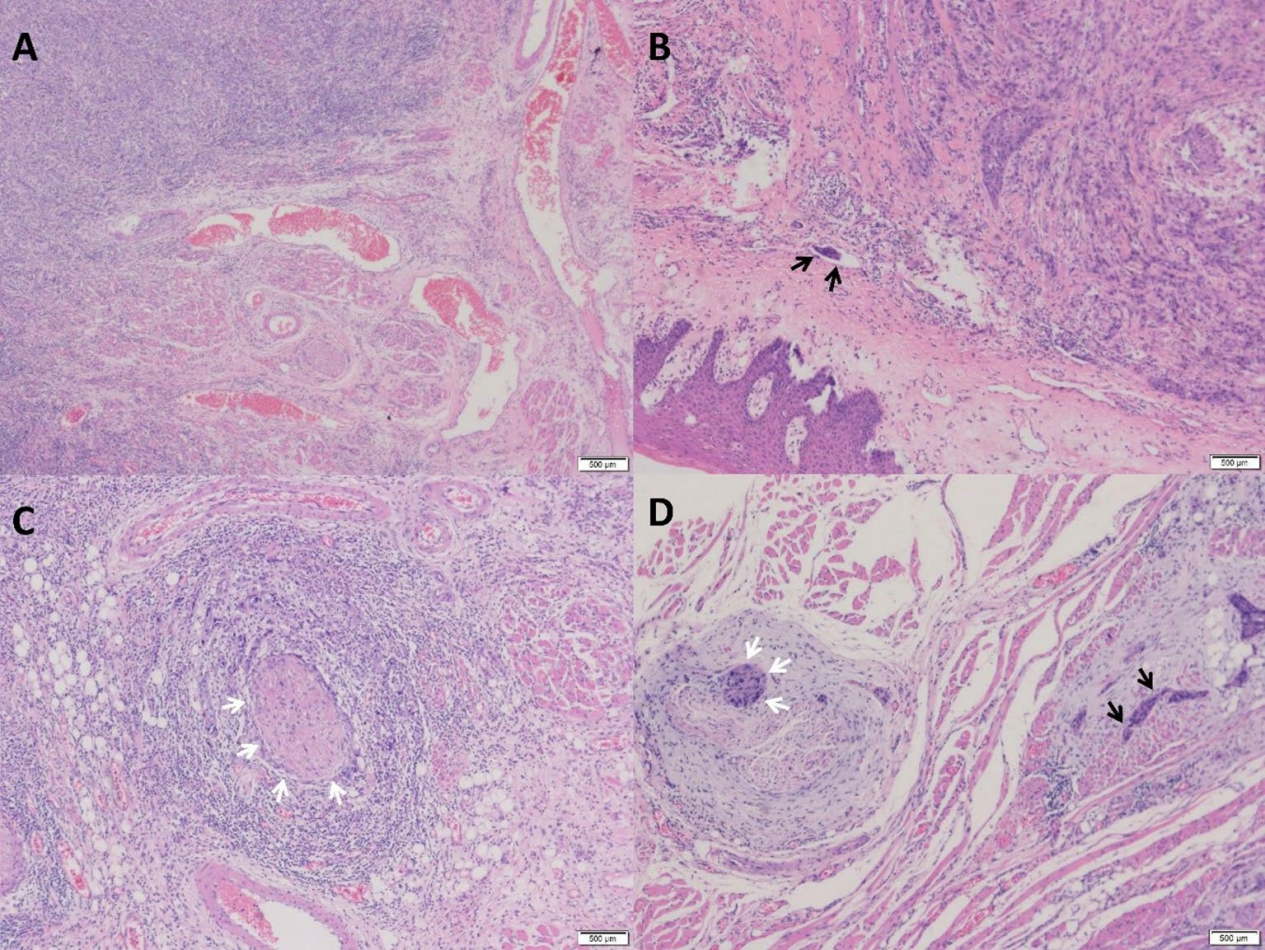
Pathological features of the four groups. Pathological sections of SCCT were stained with hematoxylin and eosin. (**A**) P–V−, PNI negative with LVI negative (× 20); (**B**) P–V + , PNI negative with LVI positive (× 40); (**C**) P + V−, PNI positive with LVI negative (× 40); (**D**) P + V + , PNI positive with LVI positive (× 40). The black arrow indicates tumor cells invading the blood and lymphatic vessels. The white arrow indicates tumor cells invading the nerves. *SCCT* squamous cell carcinoma of the tongue, *PNI* perineural invasion, *LVI* lymphovascular invasion.

Among the 127 patients who were included, 95 patients (74.8%) were classified as P–V−, 8 (6.3%) were P–V + , 18 (14.2%) were P + V−, and 6 (4.7%) were P + V + (Fig. 3A–D). The mean follow-up time was 41 months (range, 3–111 months), and 33 patients died during the follow-up period. The 1-year (χ^2^ = 11.139, p = 0.011), 3-year (χ^2^ = 29.237, p < 0.001), and 5-year survival rates (χ^2^ = 7.664, p = 0.022) were significantly different among the four groups (Table S1).

### Cox proportional hazard modeling for LVI and PNI 

Univariate analysis was used to evaluate the prognostic significance of PNI, LVI, and other pathological factors. In univariate analysis, the OS rate was negatively influenced by six tumor-related factors: pN stage, tumor stage, histological grade, postoperative radiotherapy, PNI, and LVI. Sex, age, and pT stage were not risk factors for OS (Fig. S2). The results of multivariate analysis showed that PNI (HR = 3.309; 95% CI 1.466–7.472; p = 0.004) and LVI (HR = 4.537, 95% CI 1.871–11.001; p = 0.001) were significantly correlated with OS and were independent risk factors for the prognosis of SCCT. The univariate and multivariate analyses of LVI, PNI, and various factors are shown in Table 2.

**Table 2:** Univariate and multivariate analysis of LVI, PNI and other related factors.

	Overall survival			
	Univariate analysis		Multivariate analysis	
	HR (95% CI)	p-value	HR (95% CI)	p-value
Gender				
Female		0.66		
Male	1.168 (0.585–2.329)			
Age (years)				
< 60		0.947		
≥ 60	1.024 (0.513–2.043)			
Smoking				
No		0.293		
Yes	0.622 (0.257–1.508)			
Alcohol				
No		0.702		
Yes	0.849 (0.369–1.957)			
Pathologic T stage				
T1-2		0.144		
T3-4	1.74 (0.827–3.661)			
Pathologic N stage				
0		< 0.001		0.452
1	3.117 (1.32–7.364)		1.103 (0.255–4.773)	
2	6.108 (2.728–13.677)		1.954 (0.459–8.319)	
Tumor stage				
I-II		0.001		0.821
III-IV	3.303 (1.622–6.725)		1.165 (0.311–4.356)	
Histological grade				
Poorly		0.006		0.058
Moderately	5.116 (1.381–18.954)		2.281 (0.568–9.16)	
Well	3.136 (1.435–6.856)		0.861 (0.191–3.879)	
Postoperative radiotherapy				
No		0.037		0.193
Yes	2.263 (1.051–4.872)		1.739 (0.756–3.999)	
LVI				
Negative		< 0.001		0.001
Positive	7.56 (3.645–15.684)		4.537 (1.871–11.001)	
PNI				
Negative		< 0.001		0.004
Positive	3.497 (1.731–7.063)		3.309 (1.466–7.472)	

### Hierarchical analysis of LVI and PNI

**Figure 4:**
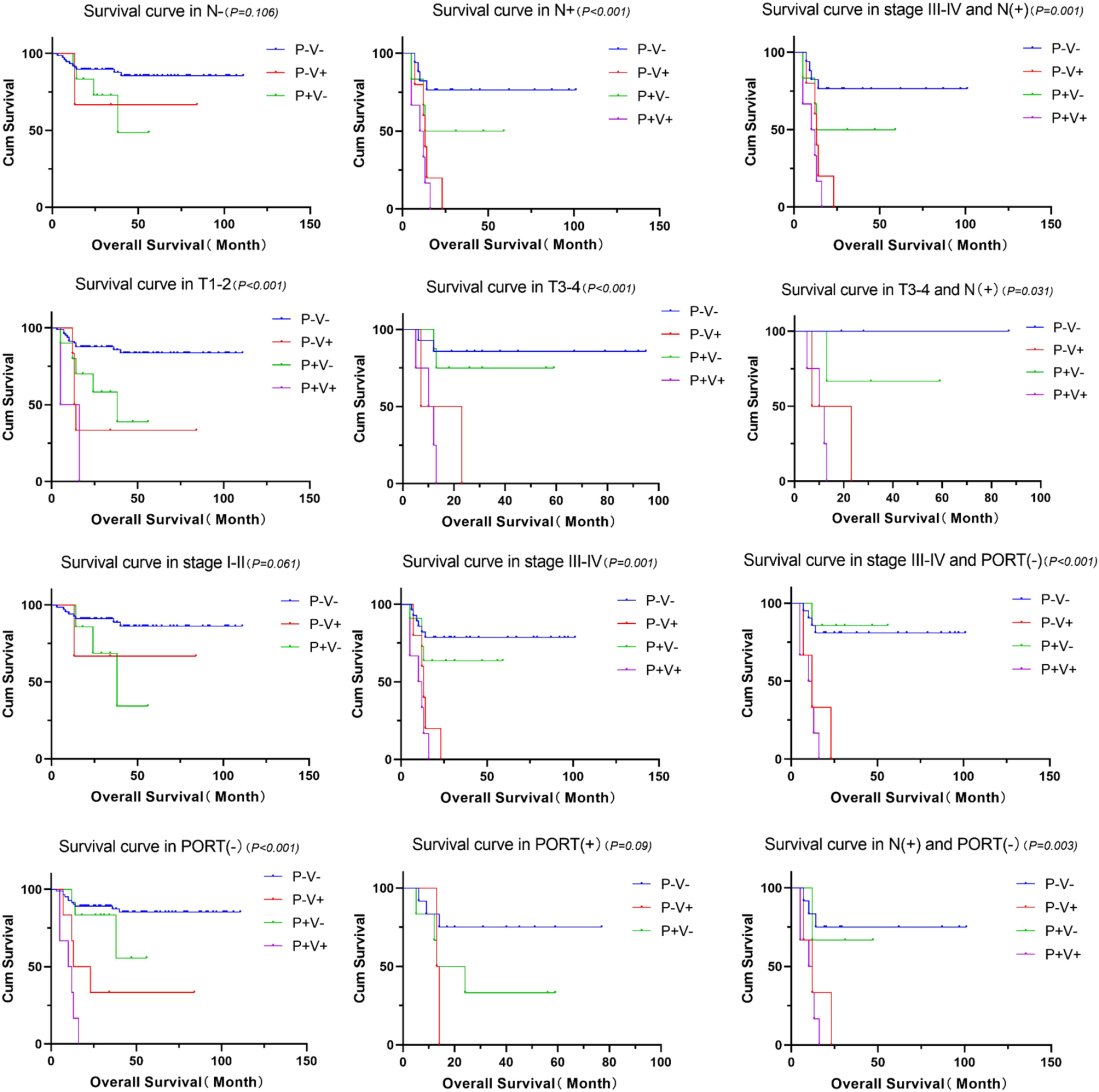
Associations of LVI and PNI with OS in different situations and subgroup comparisons. *PNI* perineural invasion, *LVI* lymphovascular invasion, *OS* overall survival.

The pT stage (T1–T2 vs. T3–T4), pN stage (N0 vs. N +), tumor stage (I–II vs. III–IV), and histological grade were analyzed. Significant differences were detected in the OS rates of the four groups at T1–T2 and T3–T4, and the OS in the P–V− group was significantly higher than that in the other three groups. No statistical difference was noted in the OS rates among the four groups in stages I–II, whereas statistical differences were detected in stages III–IV, with the OS in the P–V− group being the highest and that in the P + V + group being the lowest. Significant differences were found between the four groups in moderate and good differentiation. A significant difference was observed in the OS of the four groups with positive lymph nodes. In cases with positive lymph nodes, the OS rates in the four groups in T1–T2 and T3–T4 were compared, and the results showed statistical differences in T3–T4 between the four groups, and the OS rates in the P–V + , P + V−, and P + V + groups were significantly poorer than that in the P–V− group. Similarly, the OS rates in the four groups was analyzed, in which lymph node metastasis occurred in patients with stages III–IV. The results showed significant differences in the OS rates among the four groups in stages III–IV combined with positive lymph nodes. The OS in the P + V + group was the lowest, whereas no significant difference was detected in cases with negative lymph nodes (p = 0.979). Figures 4 and S3 show the relationships of LVI and PNI with OS in different situations.

### Survival time for the four groups

The median survival time was 47 ± 30.1 months (95% CI 41.1–53.3) in the P–V− group, 25 ± 25.3 months (95% CI 3.9–46.1) in the P–V + group, 29 ± 16.6 months (95% CI 20.5–37.1) in the P + V− group, and 10 ± 4. 4 months (95% CI 5.5–14.8) in the P + V + group. The results showed that the P + V + group had a shorter survival time (only 10 months on average). The OS for P + V− patients was longer than that for P–V + patients but shorter than that for P–V− patients. The 3-year survival rate in the P + V + group was significantly lower than that in the other three groups, whereas the 5-year survival rate in the P–V− group was significantly higher.

## Discussion

### Sex and age

The high prevalence of SCCT worldwide may be related to poor habits, such as chewing betel nuts, smoking, and alcohol consumption, which are risk factors for SCCT^27,28^. It has been reported that males are twice as likely to develop SCCT^3^. In most countries, the average age of patients with SCCT is between 51 and 55 years^27^, with an incidence of approximately 17% in patients < 40 years old^29,30^. This was consistent with our study that included 35 people < 50 years old, with a minimum age of 28 years^31^. A previous study indicated no age or sex differences in the survival rate^27^. Our study also showed that age and sex were not prognostic indicators and that there were no significant differences in LVI/PNI.

### Univariate and multivariate analysis

PNI is a common pathological finding in head and neck cancers, including squamous cell and adenoid cystic carcinomas^32^. PNI is a pathological process in which tumor cells invade the nerves and spread along the nerve sheaths, not in the form of multiple lymph or blood but as a direct extension of the primary tumor^33,34^. PNI is a histological manifestation of tumor cell infiltration^34^, which is an important cause of morbidity and mortality, and its prognosis is poor^35–37^. Cancer cell extension along nerves has been reported to increase the recurrence rate and decrease the survival rate^38^. The presence of LVI indicates that a significant number of tumor cells enter the blood vessels, which is the first step in potential metastasis^39^. In our study, pN stage, tumor stage, histological grade, PORT, PNI, and LVI were risk factors for poor prognosis. The results of the multivariate analysis showed that PNI/LVI was significantly correlated with OS and was an independent risk factor for SCCT prognosis.

### LVI, PNI, and their correlation with clinicopathologic parameters

Recent studies have shown that PNI is more common in advanced tumors, thus suggesting a poor prognosis^6^. LVI and PNI were significantly different in pT, pN, and tumor stages and were more common in T3–T4, N + , and stage III–IV patients. Many studies have consistently shown that LVI and PNI increase the risk of lymph node metastasis and that lymphatic vessel invasion is the first step in metastasis development^37,40,41^. Survival rates decreased significantly among patients with early tongue cancer when LVI was associated with other adverse prognostic factors^42^.

### Correlations of LVI and PNI with OS

PNI is considered an important prognostic factor for head and neck cancers, can be observed in the absence of lymphatic or vascular invasion, and often occurs in small and unnamed nerve fibers with an incidence of 42–52%^34,35,43^. LVI is an early indication of metastasis to lymph nodes and other organs (indicating a tendency for tumor cell invasion^17^) and is a risk factor for lymph node metastasis^44^. It has been reported that the presence of LVI and PNI suggests poor survival and can be used as prognostic biomarkers for patients with OSCC^18,45^. A strong correlation was found between LVI and survival outcomes in recurrent tumors^40^. Thus, we divided all participants into four groups according to LVI and PNI. OS in the four groups was statistically different; it was highest for P–V− and lowest for P + V + . The results showed that the P + V + group had a shorter survival time (only 10 months on average). Patients with P + V- had a longer OS than those with P–V + but had a shorter OS than those with P–V−. The 3-year survival rate in the P + V + group was significantly lower, whereas the 5-year survival rate in the P–V− group was significantly higher than that in the other three groups. This indicates that the prognosis of simultaneous neurovascular invasion is the poorest and that the prognosis of vascular invasion alone is poorer than that of neurotransplantation.

### Hierarchical analysis of the four groups

For patients with positive lymph nodes, a significant difference in OS was found among the four groups. Therefore, when patients with SCCT develop lymph node metastases, the prognosis of P + V + is the poorest among groups. OS in the P–V− group was higher than that in the other three groups at T1–T2 and T3–T4. OS was significantly different among the four groups for stage III–IV cases, and OS in the P + V + group was poorer than the OS rates in the P–V−, P–V + , and P + V− groups. Significant between-group differences were observed in terms of moderate and good differentiation, and the P + V + group showed the poorest prognosis for patients with moderately differentiated tumors. Patients in T3–T4 with lymph node metastases in the P–V + , P + V− and P + V + groups have poorer prognoses than those in the P–V− group. Patients with SCCT with both PNI and LVI have the poorest prognosis if lymph node metastases occur in stages III–IV.

### Postoperative radiotherapy and prognosis

Radiation therapy is known to be effective for oral cancer because it provides a high dose of radiation locally while preserving the surrounding healthy tissue^46^. The 2016 National Comprehensive Cancer Network guidelines recommend adjuvant radiation therapy when PNI and/or LVI are diagnosed after surgery^47^. Some studies have shown that appropriate postoperative radiotherapy can improve local control for patients with LVI and who were PNI positive^16,18,34^. Postoperative radiotherapy is recommended for patients with adverse features (stage IV), positive margins, LVI, and PNI^48–50^. Previous research has shown that PORT does not provide significant survival benefits in patients with stage I–II OSCC with PNI and/or LVI as the sole risk factor and may not be recommended^51^. In the current study, patients receiving postoperative radiotherapy generally had advanced lymph node metastases, which also predicted a poor prognosis. According to a relevant report, adding chemotherapy to radiotherapy for high-risk locally advanced head and neck cancer improved local control rates and OS^52^. For patients with stage III–IV disease not receiving PORT, there was a significant difference in survival among the four groups, with OS being the highest in the P + V− group. Therefore, a precise conclusion on whether patients with SCCT with LVI/PNI should receive radiation therapy after surgery could not be reached. Further studies are warranted to address this issue.

### Limitations

First, this study was limited by its retrospective design, including selection bias and the lack of medical record data. Patients deemed suitable for surgery were included in the analysis, which may have also yielded selection bias. Second, further analyses of LVI and PNI may be particularly undermotivating to achieve significant results because of the limited number of patients in this subgroup. Third, patients receiving postoperative radiotherapy are mainly concentrated in advanced or lymph node metastases, thus limiting further studies on radiation therapy. Finally, due to the absence of available data, further analysis of disease-specific survival cannot be achieved in the present research. Future studies should be focused on this issue.

## Conclusion

LVI and PNI are independent negative prognostic factors for patients with SCCT. Patients with LVI and/or PNI had a significantly lower OS than those without neurovascular involvement. The classification of patients with SCCT based on the TNM staging system combined with LVI and PNI may be more comprehensive and accurate. Future studies with larger sample sizes are warranted to confirm our findings, preferably with prospective designs.

## Supplementary Information

Supplementary Information.

## Data Availability

The raw data supporting the conclusions of this article will be made available by corresponding authors(Chaohui Zheng), without undue reservation.

## Abbreviations

AJCC: American joint commission on cancer
LVI: Lymphovascular invasion
OSCC: Oral squamous cell carcinoma
OS: Overall survival
PORT: Postoperative radiotherapy
PNI: Perineural invasion
SCCT: Squamous cell carcinoma of the tongue
