# Supplementary material for: Prognostic impact of lymphovascular and perineural invasion in squamous cell carcinoma of the tongue
Source: Sci Rep. 2023 Mar 7;13:3828. doi: 10.1038/s41598-023-30939-8 (PMC9992656; doi:10.1038/s41598-023-30939-8)

Prognostic Impact of Lymphovascular and Perineural Invasion in Squamous Cell Carcinoma of the Tongue

Qiongling Huang[1], Yanjun Huang[1], Chunhui Chen[2], Yizheng Zhang[1], Jiao Zhou[1], Chengke Xie[3], Ming Lu[1], Yu Xiong[2], Dage Fang[4], Yubin Yang[4], Weipeng Hu[2,*], Feng Zheng[2,*], Chaohui Zheng[1,*]

Table S1. Survival rate in the four groups

	P-V-(n=95)	P-V+(n=8)	P+V-(n=18)	P+V+(n=6)	χ^2	p-value
1-year survival rate	91.60%	87.50%	94.40%	50%	11.139	0.011
3-year survival rate	85.70%	16.70%	55.60%	0%	29.237	<0.001
5-year survival rate	82.90%	0%	0%	0%	7.664	0.022

Figure S1. Flow chart of patient inclusion.

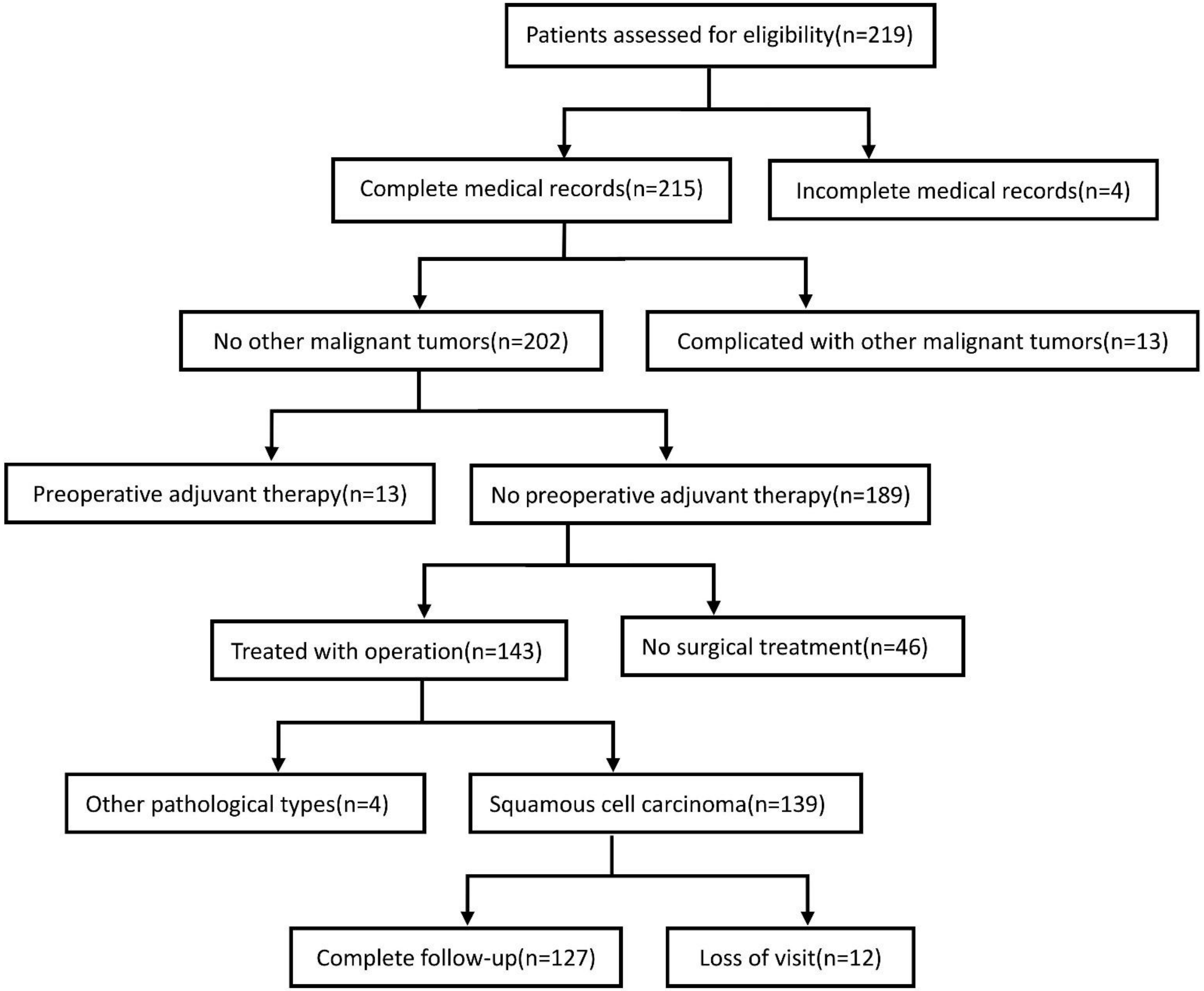

Figure S2. The relationship between the factors and the OS

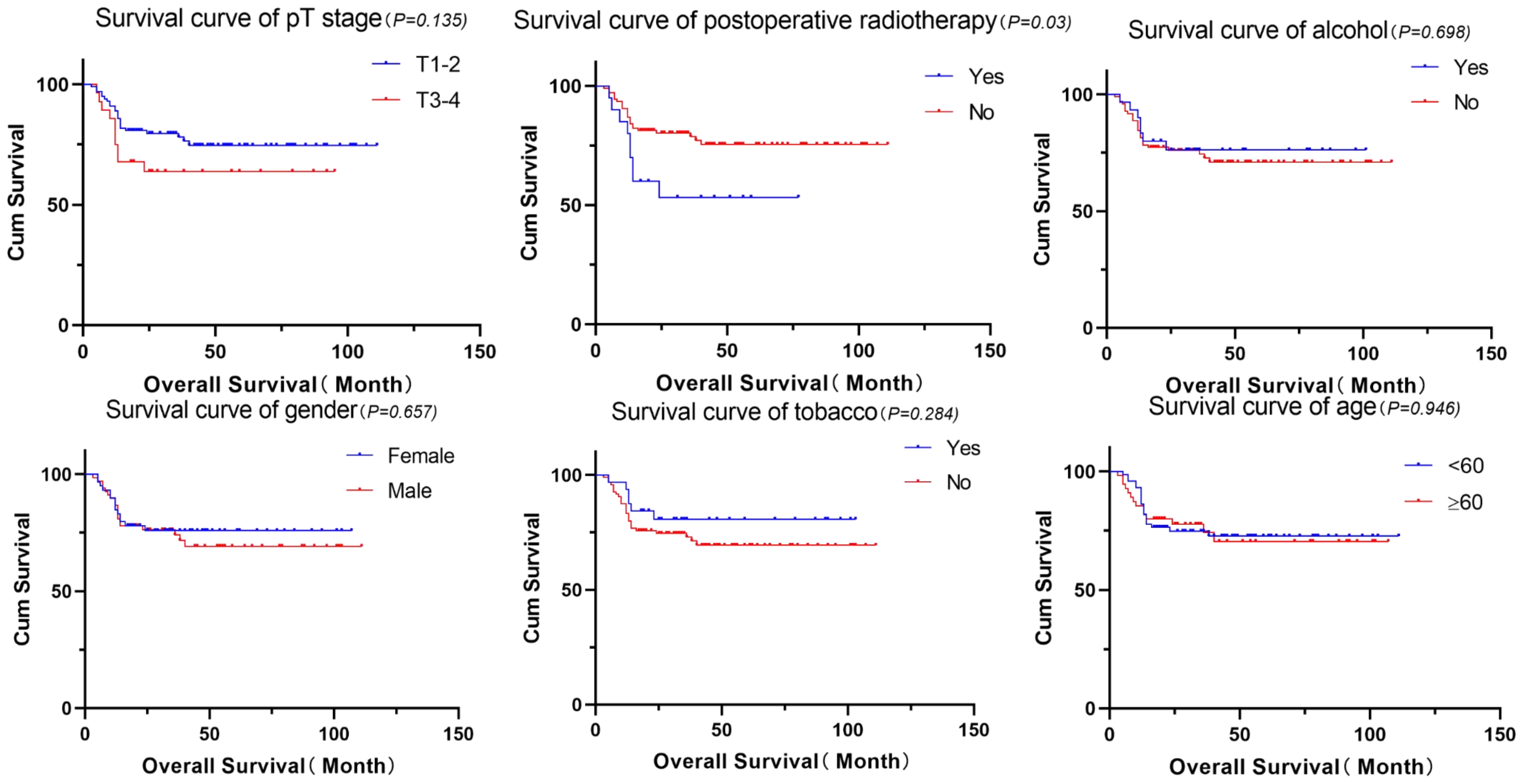

Figure S3. The relationship between LVI, PNI and the OS in different situations and subgroup comparison

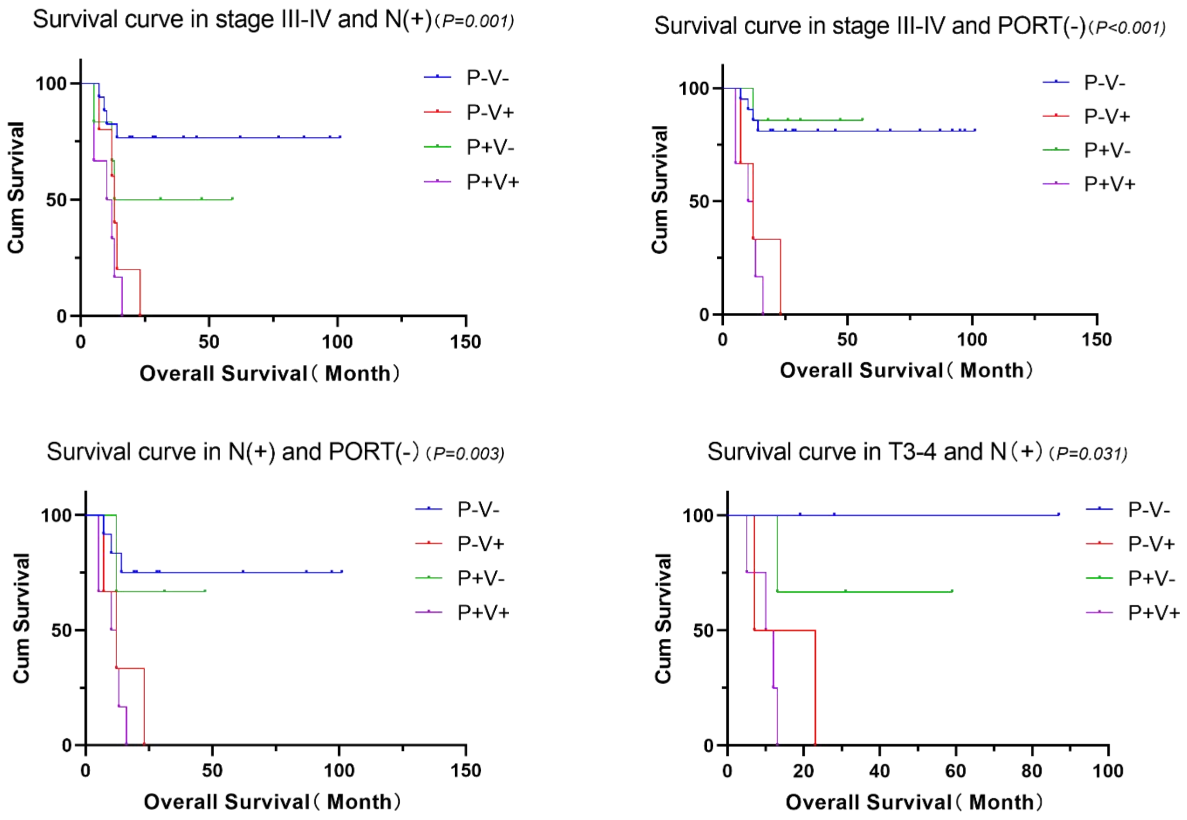

Supplement: Supplementary file 1 — Supplementary Information. [file 41598_2023_30939_MOESM1_ESM.pdf]
